# Novel polysome messages and changes in translational activity appear after induction of adipogenesis in 3T3-L1 cells

**DOI:** 10.1186/1471-2199-13-9

**Published:** 2012-03-21

**Authors:** Carolin Fromm-Dornieden, Silvia von der Heyde, Oleksandr Lytovchenko, Gabriela Salinas-Riester, Bertram Brenig, Tim Beissbarth, Bernhard G Baumgartner

**Affiliations:** 1 Institute of Veterinary Medicine, University of Göttingen, Burckhardtweg 2, 37077 Göttingen, Germany; 2 Statistical Bioinformatics, Department of Medical Statistics, University Medical Center, Humboldtallee 32, 37073 Göttingen, Germany; 3 DNA Microarray Facility Göttingen, Department of Developmental Biochemistry, University of Göttingen, Humboldtallee 23, 37073 Göttingen, Germany; 4 Department of Internal Medicine, Metabolic Diseases and Medical Molecular Biology, Paracelsus Private Medical University Salzburg, Müllner Hauptstr. 48, 5020 Salzburg, Austria

## Abstract

**Background:**

Control of translation allows for rapid adaptation of the cell to stimuli, rather than the slower transcriptional control. We presume that translational control is an essential process in the control of adipogenesis, especially in the first hours after hormonal stimulation. 3T3-L1 preadipocytes were cultured to confluency and adipogenesis was induced by standard protocols using a hormonal cocktail. Cells were harvested before and 6 hours after hormonal induction. mRNAs attached to ribosomes (polysomal mRNAs) were separated from unbound mRNAs by velocity sedimentation. Pools of polysomal and unbound mRNA fractions were analyzed by microarray analysis. Changes in relative abundance in unbound and polysomal mRNA pools were calculated to detect putative changes in translational activity. Changes of expression levels of selected genes were verified by qPCR and Western blotting.

**Results:**

We identified 43 genes that shifted towards the polysomal fraction (up-regulated) and 2 genes that shifted towards free mRNA fraction (down-regulated). Interestingly, we found Ghrelin to be down-regulated. Up-regulated genes comprise factors that are nucleic acid binding (eIF4B, HSF1, IRF6, MYC, POLR2a, RPL18, RPL27a, RPL6, RPL7a, RPS18, RPSa, TSC22d3), form part of ribosomes (RPL18, RPL27a, RPL6, RPL7a, RPS18, RPSa), act on the regulation of translation (eIF4B) or transcription (HSF1, IRF6, MYC, TSC22d3). Others act as chaperones (BAG3, HSPA8, HSP90ab1) or in other metabolic or signals transducing processes.

**Conclusions:**

We conclude that a moderate reorganisation of the functionality of the ribosomal machinery and translational activity are very important steps for growth and gene expression control in the initial phase of adipogenesis.

## Background

White adipose tissue (WAT) plays an important role in homeostasis by storing and releasing triglycerides and by releasing many hormonal factors called adipokines such as leptin or adiponectin. High caloric intake leads to the expansion of WAT by an increase of adipocyte numbers (hyperplasy), by expanding the volume of existing adipocytes (hypertrophy) or a combination of both [1]. In order to understand, which mechanisms drive hypertrophic or hyperplastic obesity, we need to understand the mechanisms controlling growth and differentiation of adipocytes.

Many factors have already been identified, mainly by RNA analysis, which regulate cell fate and control the transformation of preadipocytes into mature adipocytes. This process confers massive changes in the structure of the cell, which leads to storage of fat and the formation of fat vacuoles. The orchestrated changes in the structure of the preadipocyte require tight control of the involved factors.

An extensively studied model for adipogenesis *in vitro *is the mouse embryonic fibroblast cell line 3T3-L1 [2-4]. 3T3-L1 cells are grown to confluency and among stimulation with a hormone cocktail consisting of insulin, dexamethasone and isobutylmethylxanthine (IBMX), cells re-enter cell cycle within 24 to 36 h. Two rounds of cell division are performed after which they permanently withdraw from the cell cycle, begin to accumulate lipid, and undergo terminal differentiation into mature adipocytes. Until recently, regulating mechanisms of the earliest step in the differentiation process are poorly understood in comparison to later stages of adipogenesis.

The orchestrated changes in the expression of structural proteins require changes in the expression and activity of factors controlling the expression of structural proteins. Induction of adipogenesis by hormones leads to the expression of C/EBPβ and C/EBPδ within 2 hours [5], which activate the expression of *PPARγ *and *C/EBPα *at low levels. PPARγ and C/EBPα activate each others' expression in a positive feedback loop that promotes and maintains the differentiated state [6]. The latter proteins orchestrate the changes in gene expression typical for mature adipocytes. Besides the C/EBP family and PPARγ, many other factors are known that play a role in adipogenesis. Most of these factors are regulated on the transcriptional level and mRNA steady state levels can be easily measured. However, there is increasing evidence that proteins, which are controlled at the translational level, are of uttermost importance for adipogenesis and other cellular processes. A handful of translationally controlled proteins has been characterized in adipogenesis up to now. The C/EBP factors C/EBPα and C/EBPβ are regulated at the posttranscriptional level, whereupon various isoforms arise from a unique mRNA by differential initiation of translation [7]. In 3T3-L1 cells, high eIF2 and eIF4E activity shifts the ratio of C/EBP isoforms expression toward more truncated isoforms [7]. An important factor that controls adipogenesis at the translational level is HZF [8]. HZF enhances translation of *C/EBPα *mRNA by binding to its 3´-untranslated region. Knock-down of HZF disrupts adipogenesis.

In most cases, translation efficiency is closely related to ribosomal association of the respective mRNA. The degree of ribosomal association can be easily measured by separation of the mRNAs in a sucrose gradient by velocity sedimentation [9]. The gradient is fractionated, mRNA is isolated from each fraction and the ratio of the 28S/18S rRNA is used to determine the fractions containing free RNA (no ribosomes bound) and polysomal RNA [10]. By use of microarrays, the composition of the RNA pools can be determined easily [11,12].

We used velocity sedimentation and microarray analysis to identify mRNAs that change in ribosomal association during the first 6 hours after hormonal induction of adipogenesis of 3T3-L1 cells and assessed changes of translational activity by Western blotting of some candidate genes.

## Results

### Adipogenesis

Adipogenesis was assessed microscopically by Oil Red O staining (Additional file 1). In comparison to the fibroblastic phenotype of preadipocytes, mature adipocytes´ phenotype is round and cells accumulate lipid droplets in the cytoplasm. Additionally, adipogenesis was assessed by the analysis of mRNA steady state levels of *C/EBPβ *and *PPARγ *by means of q-PCR in total RNA at time points day 0, 1 and 9. Ct-values were calibrated to day 0, normalized with *β*Actin, (mean of 3 experiments with 3 replicates each, n = 9). *C/EBPβ *was up-regulated 3times at day 1 and back to base levels at day 9. *PPARγ *was up-regulated at day 9, no significant change of mRNA steady state levels were detected at day 1 (Additional file 2). These data confirm correct adipogenesis [13].

### Velocity sedimentation and polysome analysis

We analyzed mRNA distribution in samples taken immediately before (0 h) and 6 hours after hormonal stimulation (0 h + 6). 13 fractions of 1 ml each were collected from the top of the ultra centrifuged gradient. Stability of linear gradients was proved by refractive index of sucrose concentration (Additional file 3). The gradients showed high stability in fraction sucrose concentration. After RNA isolation, the ratio of 28S to 18S rRNA was measured to determine the distribution of free RNA and polysomal RNA. Non-polysomal RNA shows 28S/18S ratios unequal to two, while polysomal fractions show 28/18S ratios close to two [10]. Fractions 5, 6 and 7 were pooled and considered as free RNA (Additional file 4). Fractions 9, 10 and 11 were pooled and considered as polysomal RNA. Fraction 1 to 4 contained mainly buffer, remaining cell lysate and fat, fraction 12 and 13 contained cell debris. These fractions were not used for ribosome profiling. Fraction 8 was not used in order to obtain a clear separation of polysomal and non-polysomal fractions.

### Microarray analysis

**Figure 1:**
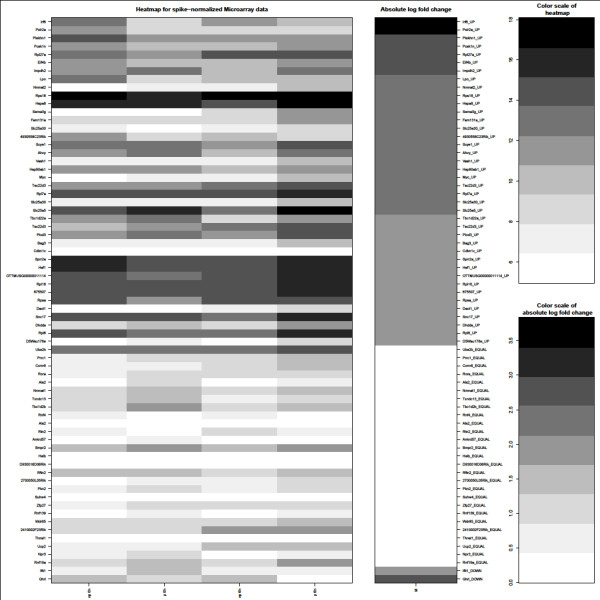
**Heatmap for spike-normalized microarray expression data**. Columns 1 to 4 show log2 expression data for polysomal (p) and non-polysomal (np) fractions at two time points (0 h and 6 h after hormonal induction). Column 5 shows log2 fold change M ((p6-np6) - (p0-np0)). Only genes with fdr <0.05 were selected. Genes were identified as up-regulated (Up; highest expression in polysomal fractions at time point 6 h after hormonal induction) with M-value > 2, p6 > p0, p6 > np6, np6 < np0, p0 < np0. Genes were identified as down regulated (Down) with M-value < -2, p6 < p0 and as neither up nor down regulated (Equal) with equivalence test parameter 'epsilon = 0,2'. High expression levels are shown in dark grey, low expression levels in white.

Microarray analysis was performed according to standard protocols in three biological replicates (Agilent Technologies; Cat. No. G4122F; 41,000 genes represented) [14]. Expression levels were normalized to the spike values and the quality control revealed the expected clustering of fractions into polysomal and non-polysomal ones as well as clustering of time points (see methods). Data were submitted to GEO (accession number GSE29744). We identified 918 up-regulated and 20 down-regulated genes with a false discovery rate (fdr) <0.05 and with M-value (=log2(fold change)) >1. To exclude false positives from decreasing total mRNA steady state levels (relative increase in polysomal RNA versus free RNA after induction) only genes with values of p_6 _> p_0_, p_6 _> np_6_, np6 < np_0_, p_0 _< np_0 _were included in the analysis. For the present study, only genes with M-value > 2 were considered. 43 genes showed highest abundance in polysomal fractions at time point 6 h after hormonal induction and were identified as up-regulated (Table 1 and Additional file 5). Two genes were identified as down-regulated with M-values < -2, p_6 _< p_0 _and p_0 _> np_0 _(Table 1 and Additional file 5). Determination of unchanged gene expression between (p_6_-p_0_) and (np_6_-np_0_) was based on two one-sided tests for equivalence gene-by-gene [15,16] in which a magnitude of 0.2 was chosen for the region of similarity and 27 genes fit these parameters (Figure 1).

**Table 1:** GenBank accession numbers for mRNAs that are fourfold and greater up- or down-regulated 6 hours after stimulation of adipogenesis (for further information, e

mRNAs up-regulated 6 hours after stimulation of adipogenesis	
AK087631	interferon regulatory factor 6 (*IRF6)*
NM_009089	polymerase (RNA) II (DNA directed) polypeptide A (*POLR2a)*
NM_001008233	pleckstrin homology domain containing, family N member 1 (*PLEKHN1)*
NM_013892	proprotein convertase subtilisin/kexin type 1 inhibitor (*PCSK1n)*
NM_011975	ribosomal protein L27a (*RPL27a)*
NM_145625	eukaryotic translation initiation factor 4B (*eIF4B)*
NM_011830	inosine 5'-phosphate dehydrogenase 2 (*IMPDH2)*
NM_080420	lactoperoxidase (*LPO)*
NM_175460	Nicotinamide nucleotide adenylyltransferase 2 (*NMNAT2)*
NM_011296	ribosomal protein S18 (*RPS18)*
NM_031165	heat shock protein 8 (*HSPA8)*
AK129018	sema domain, immunoglobulin domain (Ig), short basic domain, secreted, (semaphorin) 3G (*SEMA3g)*
NM_133778	family with sequence similarity 131, member A (*FAM131a)*
NM_026232	solute carrier family 25, member 30 (*SLC25a30)*
AK006075	RIKEN cDNA 4930558C23 gene (*4930558C23RIK)*
NM_007926	small inducible cytokine subfamily E, member 1 (*SCYE1)*
L32836	S-adenosylhomocysteine hydrolase (*AHCY)*
NM_177354	vasohibin 1 (*VASH1)*
NM_008302	heat shock protein 90 alpha (cytosolic), class B member 1 (*HSP90ab1)*
NM_031160	ADP-ribosylation factor-like 4D (*ARL4d)*
NM_010849	myelocytomatosis oncogene (*MYC)*
AF024519	TSC22 domain family, member 3 (*TSC22d3)*
NM_013721	ribosomal protein L7A (*RPL7a)*
AK159732	solute carrier family 25, member 30 (*SLC25a30)*
XR_002409	no description
NM_007451	solute carrier family 25 (mitochondrial carrier, adenine nucleotide translocator), member 5 (*SLC25a5)*
NM_145476	TBC1 domain family, member 22a (*TBC1d22a)*
NM_001077364	TSC22 domain family, member 3 (*TSC22d3)*
NM_011962	procollagen-lysine, 2-oxoglutarate 5-dioxygenase 3 (*PLOD3)*
AJ250687	BCL2-associated athanogene 3 (*BAG3)*
NM_009876	cyclin-dependent kinase inhibitor 1C (P57) (*CDKN1c)*
NM_011468	small proline-rich protein 2A (*SPRR2a)*
Z49206	heat shock factor 1 (*HSF1)*
NM_001013830	no description
XM_973351	ribosomal protein S15a pseudogene (*GM13253)*
NM_009077	ribosomal protein L18 (*RPL18)*
XR_005114	predicted gene, 675507
NM_011029	ribosomal protein SA (*RPSa)*
NM_021532	dapper homolog 1, antagonist of beta-catenin (xenopus) (*DACT1)*
NM_153680	sorting nexin 17 (*SNX17)*
NM_026144	dehydrodolichyl diphosphate synthase (*DHDDS)*
NM_011290	ribosomal protein L6 (*RPL6)*
NM_027652	ethanolaminephosphotransferase 1 (*EPT1)*

**mRNAs down-regulated 6 hours after stimulation of adipogenesis**	

NM_008331	interferon-induced protein with tetratricopeptide repeats 1 (*IFIT1)*
NM_021488	ghrelin (*GHRL)*

### Quantitative Real Time-PCR (q-PCR)

In order to assess up-regulation of genes as determined by microarray analysis, we chose three up-regulated genes (*RPL27a, eIF4B *and *IMPDH2*) and one gene with unchanged mRNA (*UBE2k/HIP2*) for target verification by q-PCR. The RNA preparation used for q-PCR was the same as the one used for Agilent 2100 Bioanalyzer-analysis. q-PCR data confirmed microarray results. *RPL27a *was up-regulated 7.4 times in microarray analysis (MA) (fdr: 2.38E-07), and 13.8 times up-regulated in q-PCR (p-value: 0.0163). *eIF4B *was 7.2 times up-regulated in MA (fdr: 4.12E-07) and 8.3 times up-regulated (p -value: 0.1423) in q-PCR. *IMPDH2 *was 6 times up-regulated in MA (fdr: 3.26E-07) and 12.2 times up-regulated (p-value: 0.073) in q-PCR. *UBE2k *was neither up nor down-regulated in MA (fdr: 0.013) or q-PCR. Hence, up-regulation of three randomly chosen genes was successfully confirmed by q-PCR (Additional file 6).

### Western Blotting

**Figure 2:**
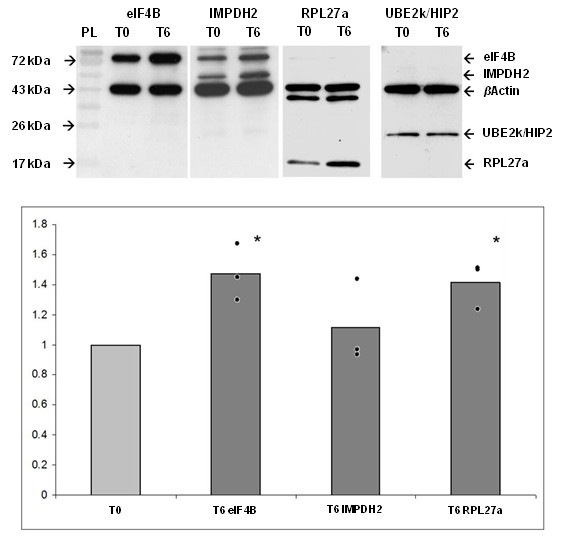
**Western blot results**. 3T3-L1 cells were differentiated with insulin, dexamethasone and IBMX. Protein was isolated from whole cell extracts 0 h (T0) and 6 h (T6) after hormonal induction. 30 μg of each sample was subjected to Western blot analysis for eIF4B, IMPDH2, RPL27a and UBE2k/HIP2 expression. Changes in protein expression were quantified by densitometry and normalized with appropriate expression data of *β*Actin. The values represent the average of three independent experiments, and the asterisk denotes a p-value < 0.05. eIF4B and RPL27a protein expression is 1.4 fold higher (p-value < 0.05) at T6 than time point T0. IMPDH2 and UBE2k/HIP2 show no significant differences in protein expression between T0 and T6.

To confirm up-regulation of protein levels of mRNAs identified in microarray assays and confirmed by q-PCR, 3T3-L1 cells were differentiated for 6 hours and protein levels were analyzed by Western blotting. We analyzed protein levels of the three up-regulated proteins *eIF4B, IMPDH2 *and *RPL27a *and the unchanged gene *UBE2k *in three biological replicates, each biological replicate being performed twice. Changes in protein expression were quantified by densitometry and normalized with appropriate expression data of *β*Actin (Figure 2). eIF4B and RPL27a protein levels were 1.4 fold higher (t-test, p-value < 0.05) at 0 + 6 h than at 0 h. Impdh2 and UBE2k/HIP2 showed no significant differences in protein levels between 0 h and 0+ 6 h after hormonal induction.

### Cluster analysis

We used PANTHER DB to classify genes into functional groups. For 5 of the 43 up-regulated mRNAs, no function is known. The known genes code for proteins that are nucleic acid binding - either RNA or DNA - (eIF4B, HSF1, IRF6, MYC, POLR2a, RPL18, RPL27a, RPL6, RPL7a, RPS18, RPSa, TSC22d3), form part of ribosomes (RPL18, RPL27a, RPL6, RPL7a, RPS18, RPSa), act on the regulation of translation (eIF4B) or transcription (HSF1, IRF6, MYC, TSC22d3), as chaperones (BAG3, HSPA8, HSP90ab1), nucleotidyl transferase (NMNAT2, POLR2a), amino acid transporter (SLC25a5, SLC25a30), kinase inhibitor (CDKN1c), aminoacyl-tRNA synthetase (SCYE1), peroxidase (LPO), oxidoreductase (IMPDH2, LPO, PLOD3), oxygenase (PLOD3) or hydrolase (AHCY, TBC1d22a) and peptide hormone (GHRL). PANTHER informations are shown in Additional file 7.

## Discussion

We analyzed the changes in the abundance of mRNAs in free or ribosome bound fractions from velocity sedimentation during the first hours of adipogenesis in 3T3-L1 mouse cells. We identified 43 genes that were significantly increased in the polysomal fraction at T6, and two genes with lower abundance (Table 1) 6 hours after the induction of adipogenesis by administration of a hormone cocktail to the confluent cell culture. We confirmed MA data by q-PCR of three randomly chosen up-regulated genes. Finally, we analyzed protein levels of the three up-regulated genes *eIF4B, IMPDH2 *and *RPL27a *and the unchanged gene *UBE2k/HIP2 *(Figure 2). In Western blots, IMPDH2 protein levels were unchanged. It is subject to further investigation, if ribosomes on *IMPDH2 *mRNA are stalled, protein levels increase later in the course of adipogenesis or if other mechanisms are employed to keep the protein levels unchanged. eIF4B and RPL27a levels were confirmed to be up-regulated and we assume that as a general rule, changes in translational activity are predicted by changes of ribosomal association as has been demonstrated before [17].

There are several studies of transcriptional changes during adipogenesis and many factors have been identified that play an important role in this process. Burton *et al.*, 2002 identified 286 clones with a greater than fivefold deviation of expression during the first 24 hours of adipogenesis [5]. In our study, none of these factors showed changes greater than fourfold with the exception of c-Myc. We conclude that analysis of translational activity is important for a full understanding of the processes controlling adipogenesis.

In the last few years, interest increased in the dynamics of the proteome and there is a handful of studies in man and mouse [18-24] about changes of the proteome, comparing preadipocytes and adipocytes in tissues, or later stages of adipogenesis. Here, we report changes as soon as 6 hours after hormonal induction of adipogenesis, a timepoint, at which clonal expansion of 3T3-L1 cells is induced and expression of mitotic and adipogenic genes is initiated.

**Figure 3:**
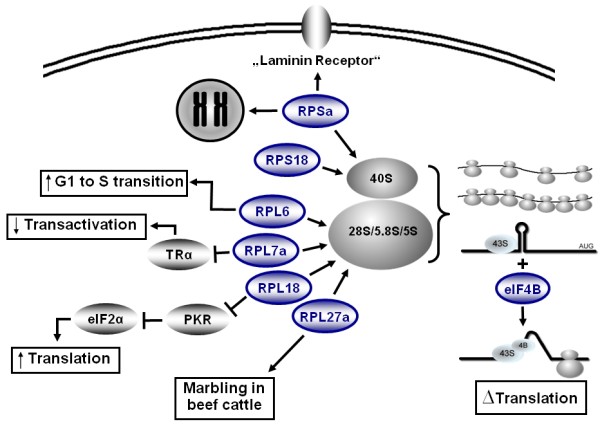
**Ribosomal and extraribosomal functions of the ribosomal proteins up-regulated in this study**. Microarray results of polysomal fractions from 3T3-L1 cell lysate (6 h after hormonal induction) show that that ribosomal proteins (RP) are prominent among the up-regulated genes. For some RPs, extraribosomal functions have been demonstrated. In the nucleus, RPSa binds to DNA by histone binding, in the cytoplasm it is associated with the 40S small ribosomal subunit and at the cell surface it acts as a receptor for various components [34]. RPL6 over-expression promotes G1 to S phase transition of gastric cancer cells and promotes cell growth [28]. RPL7a interacts with the human thyroid hormone receptor and inhibits transactivation. Hyperthyroidism favours osteosarcoma cell growth and down-regulation of RPL7a might enhance sensitivity to TR and disrupt growth control [61]. RPL18 was shown to inhibit autophosphorylation of the double-stranded RNA-activated protein kinase (PKR) and PKR mediated phosphorylation of the translation initiation factor eIF2α. Over-expression of RPL18 reduced eIF2α phosphorylation and stimulated translation of a reporter gene in vivo [31]. A polymorphism in the promoter region of the RPL27a gene was associated with meat marbling in Japanese Black beef cattle [32]. These known extraribosomal functions might be important in early adipogenesis. Additionally an enhanced amount of RPs promotes an increase in translation process of adipocyte specific genes. In the beginning of translational process, the 43S ribosomal subunit scans mRNAs for start codons. Strong secondary structures inhibit processing of the complex on the mRNAs. eIF4B increases the helicases activity of the complex and allows translation mRNAs with strong secondary structures in the 5´UTR.

3T3-L1 adipocytes are grown to confluency and upon stimulation with a hormone cocktail (insulin, dexamethasone and IBMX), post-confluent G_0 _cells reenter one to two rounds of cell-cylce called mitotic clonal expansion (MCE) [25]. It has been proposed that MCE might facilitate the DNA remodeling for the adipogenesis gene expression program [2]. As 3T3-L1 cells reenter cell cycle by passing from G_0 _to G_1 _phase, it might be expected that the translation machinery is activated after hormonal stimulation. In fact, we detected a shift of mRNAs toward the higher molecular weight polysomal fractions, mostly derived from a general activation of gene expression. Expression of ribosomal proteins, ribosome assembly proteins and ribosomal RNA (rRNA) are up-regulated in mitotic active cells. Hence it is not surprising that ribosomal proteins (RP) are prominent among the up-regulated proteins (compare Table 1). Additionally, for some RPs, extraribosomal functions have been demonstrated. Many of these extraribosomal functions can be attributed to the regulation of cell cycle and for several RPs a role in cancer, promotion of cell growth or differentiation has been shown [26]. Some of these known extraribosomal functions might well explain the role of the RPs in the early phase of adipogenesis (Figure 3).

*RPL6 *was initially identified as up-regulated in gastric multidrug-resistant cancer cells [27] and was shown to protect gastric cancer cells from drug-induced apoptosis. Furthermore, *RPL6 *over-expression promotes G1 to S phase transition of gastric cancer cells and promotes cell growth [28] (Figure 3). RPL7a interacts with the human thyroid hormone receptor and inhibits transactivation. Thyroid hormone signalling stimulates adipogenesis [29,30] and up-regulation of RPL7a might participate in mitotic control. RPL18 was shown to inhibit autophosphorylation of the double-stranded RNA-activated protein kinase (PKR) and PKR mediated phosphorylation of the translation initiation factor eIF2α. Over-expression of *RPL18 *reduced eIF2α phosphorylation and stimulated translation of a reporter gene *in vivo *[31]. Over-expression of *RPL18 *is thought to promote protein synthesis and cell growth through inhibition of PKR activity [31], which also might hold true for adipogenesis. A polymorphism in the promoter region of the *RPL27a *gene was associated with meat marbling (accumulation of intramuscular fat) in Japanese Black beef cattle [32]. Furthermore, RPL27a is ubiquitinated in a cell-cycle specific manner, leading to increased translational efficiency of the ribosomes [33].

RPSa, which was up-regulated in our study, was previously known as 37-kDa laminin receptor precursor/67-kDa laminin receptor (LRP/LR). It has a number of functions depending on its subcellular localisation. In the nucleus, RPSa binds to DNA by histone binding, in the cytoplasm it is associated with the 40S small ribosomal subunit and at the cell surface it acts as a receptor for various components [34]. It confers an anti-obesity effect when stimulated by the green tea catechin EGCG [35]. Interestingly, RPSa inhibits insulin stimulation of 3T3-L1 mitogenesis and EGCG inhibited differentiation of preadipocytes to adipocytes [36].

Most of the RPs up-regulated immediately after hormonal induction were shown to stimulate cell cycle which is concordant with the fact that 3T3-L1 cells undergo mitosis after stimulation. Translational control allows for rapid changes of protein redundancy and it may be speculated that proteins that initiate MCE and reprogramming of gene expression are regulated at the translational level. Therefore we suggest that the rapid increase of L6, L7a, L18, L27a, Sa, and S18 may reflect their importance of adipogenesis control in 3T3-L1 cells.

Higher translation rates require transport of amino acids, and we detected up-regulation of the amino acid transporters SLC25a5 and SLC25a30. Higher translation rates also lead to increased misfolding of nascent polypeptide chains. Up-regulation of chaperones in translation promoting conditions has been described before and was also observed in our study (BAG3, HSPA8, HSP90ab1).

**Figure 4:**
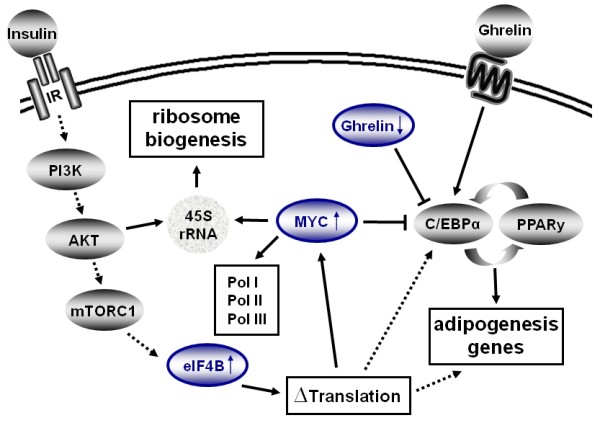
**Schematic overview of the pathway controlling translational changes in adipogenesis**. The PI3K/AKT/mTORC1 pathway, which is stimulated by insulin, leads to activation of eIF4B, which changes preferences in translation activity [38]. Regulation of C/EBPα could possibly be explained by up-regulation of eIF4b activity, as members of the C/EBP family are regulated at the translational level (dashed line). Additionally an increase in translation of adipogenesis genes mediated by eIF4B is thinkable (dashed line). c-MYC over-expression in cycling cells has been reported to block exit from the cell cycle, accelerate cell division, and increase cell size (reviewed in [45]). When c-MYC levels are high, 3T3-L1 adipoblasts are locked in a proliferation-competent state and normal differentiation can not be activated. Persisting high levels of c-MYC can inhibit the expression of genes that promote adipogenesis namely C/EBPα and PPARγ2 and therefore prevent terminal differentiation of preadipocytes to mature adipocytes [46,47]. c-MYC is an important regulator of ribogenesis, as it activates Pol I, Pol II and Pol III [49]. As a supplement in media, Ghrelin promotes the proliferation and differentiation of 3T3-L1 preadipocytes by increasing the mRNA levels of PPARγ and C/EBPα [52]. Ghrelin mRNA over-expressing 3T3-L1 cells, on the other hand, demonstrated significantly attenuated differentiation of preadipocytes into adipocytes [53]. Down-regulation of Ghrelin levels in the early phase of adipogenesis fits the known facts indicating a role of decreased endogenous Ghrelin levels in promoting adipogenesis).

The PI3K-AKT-mTOR pathway, which is stimulated by insulin, has been identified to be essential for many cellular processes (reviewed in [37]). mTORC1 is a protein complex containing mTOR (mammalian target of rapamycin) and raptor. mTORC1 activates S6K1, a kinase that promotes protein synthesis and cell growth by phosphorylation of multiple substrates including components of translation initiation or elongation such as ribosomal protein S6, eIF4B and eukaryotic elongation factor 2 kinase [38]. One target of this pathway is eIF4B (Figure 4) (reviewed in [39]). It was suggested that phosphorylation of eIF4B by S6 kinases, which are regulated by mTOR, stimulates its function. Indeed, this phosphorylation event favors recruitment of eIF4B into complexes with eIF3, which promotes the recruitment of ribosomes to the 5´end of the message (reviewed in [40]). eIF4B, which was up-regulated in our study, stimulates the RNA helicase activity of eIF4A in unwinding secondary structures in the 5´-untranslated regions (5´-UTR) of mRNAs [41,42]. By knock-down of eIF4B, selective reduction of translation was observed for mRNAs harboring strong to moderate secondary structures in their 5´-UTRs. These mRNAs code for proteins that function in cell proliferation (e.g. CDC25C, c-MYC) or cell survival (e.g. BCL-2). Silencing of *eIF4B *also leads to decreased proliferation rates and caspase-dependent apoptosis: eIF4B is required for cell proliferation and survival by regulating the translation of proliferative and prosurvival mRNAs [43]. PPARγ expression is stimulated in response to mTORC1 [44]. PPARγ is a key adipogenic factor and exogenous expression is sufficient to induce adipogenesis. Zhang *et al*., 2009 discuss the possibility that AKT and mTORC1 facilitate adipogenesis by up-regulation of PPARγ via regulation of FOXO1 [44]. However, they do not discuss the activation of eIF4B upon mTORC1 activation with subsequent changes in the preference of ribosomes for certain mRNAs. We think that regulation of C/EBPα could possibly be explained by up-regulation of eIF4b activity, as members of the C/EBP family are regulated at the translational level (Figure 4).

Microarray results of polysomal fractions from 3T3-L1 cell lysate (6 h after hormonal induction) show up-regulation of eIF4B and MYC (arrow head on top) and down-regulation of Ghrelin (arrow head below). IR, insulin receptor; Pol I/II/III, RNA polymerase I/II/III

*c-MYC *over-expression in cycling cells has been reported to block exit from the cell cycle, accelerate cell division, and increase cell size (reviewed in [45]). When c-MYC levels are high, 3T3-L1 adipoblasts are locked in a proliferation-competent state and normal differentiation can not be activated. Persisting high levels of c-MYC can inhibit the expression of genes that promote adipogenesis namely *C/EBPα *and *PPARγ2 *and therefore prevent terminal differentiation of preadipocytes to mature adipocytes [46,47]. In microarray analysis, *c-MYC *was up-regulated in 3T3-L1 cells several hours after hormonal induction in a study by Burton *et al.*, 2002 [5] and at day 2 of differentiation in a study by Kim *et al.*, 2007 [48]. c-MYC is an important regulator of ribogenesis, as it activates Pol I, Pol II and Pol III leading to activation of expression of rRNA, tRNA, ribosomal proteins, initiation factors of translation and other cell cycle relevant genes [49]. Therefore, c-MYC activation might be important for activation of the translation apparatus at the entry of 3T3-L1 cells into G_1_.

In our study, two genes were down-regulated: *IFIT1 *and ghrelin/obestatin prepropeptide (referred to as Ghrelin). The members of the *IFIT *gene family are silent in most cell types, but are activated by e.g. interferons [50]. IFIT proteins are considered silencers of translation and down-regulation might be another factor of translation stimulation.

Ghrelin has been described as a pro-adipogenic factor released by the gut and is involved in control of food intake, energy metabolism and cytokine secretion (reviewed in [51]). Treatment of 3T3-L1 preadipocytes with Ghrelin significantly increases the mRNA levels of *c-MYC*, and induces the transition from G1 to S [52]. As a supplement in media, Ghrelin promotes the proliferation and differentiation of 3T3-L1 preadipocytes by increasing the mRNA levels of *PPARγ *and *C/EBPα *[52] (Figure 4). Ghrelin mRNA over-expressing 3T3-L1 cells, on the other hand, demonstrated significantly attenuated differentiation of preadipocytes into adipocytes [53]. In the recent study we found Ghrelin ~ 6 times down-regulated in polysomal fractions of 3T3-L1 cells six hours after hormonal induction. Down-regulation of Ghrelin levels in the early phase of adipogenesis fits the known facts indicating a role of decreased endogenous Ghrelin levels in promoting adipogenesis.

## Conclusions

We have analyzed changes in translational control at 6 hours after induction of adipogenesis in 3T3-L1 preadipocytes and detected 43 translationally up-regulated and two down-regulated mRNAs with a minimal change of 4 fold. The alternatively regulated mRNAs play roles in cell cycle control, control of transcription, control of translation, energy supply, protein folding, amino acid transport and other cellular processes. A large number of RPs is up-regulated in the first hours of adipogenesis. Many RPs play a special role in cell cycle regulation and expression control of genes and mRNAs that are required for the differentiation process. Most of the nucleic acid binding factors we detected bind to RNA and play a role for ribosome function or translation control. We conclude that after stimulation of adipogenesis, translation control changes and forms a new layer of control which might be, at least in part, self-regulating. The changes in translation might come from changed properties of ribosomes in response to hormonal stimulation and from increased eIF4b activity. Further analysis of how protein levels of adipogenic genes are controlled will deepen our understanding of the complex steps during the initiation of adipogenesis and help further to develop strategies to inhibit fat cell accumulation.

## Methods

### Cells

3T3-L1 cells were ordered from HPACC and cultured in Dulbecco's modified Eagle's medium (DMEM; PAN) supplemented with 10% newborn bovine serum (c·c·pro), 25 mM HEPES (PAN) and 1% antibiotics (Penicillin/Streptomycin, c·c·pro) in 5% CO_2 _at 37°C. Absence of Mycoplasm was assessed by DAPI method on a regular basis. For differentiation, 3T3-L1 cells were cultured to confluence (day −2) and exposed to the differentiation mixture (DMEM, 10% fetal bovine serum, 25 mM HEPES, 1% antibiotics, 0.5 mM isobutylmethylxanthine, 1 μM dexamethasone, 5 μg/ml insulin; all from Sigma) two days later (day 0). After 48 hrs, medium was replaced by DMEM containing 10% fetal bovine serum, 25 mM HEPES, 1% antibiotics and 5 μg/ml insulin. At day 5, this medium was changed to medium without insulin and replaced at day 7 by fresh medium. The success of adipogenesis was proved by Oil Red O (Sigma) staining (Additional file 1) and analysis of mRNA steady state levels of well known adipogenesis factors (*C/EBPβ *and *PPARγ*) with q-PCR (Power SYBR® Green PCR Master Mix, ABI) [13], normalized with *β*Actin. Three biological replicates were used for further analysis (Additional file 2).

### Velocity sedimentation and polysome analysis

3T3-L1 cells were harvested at day 0 (T0, without hormonal induction) and 6 h after hormonal induction (T6). Cells were lysed mechanically in polysome buffer (300 mM KCl, 5 mM MgCl2, 10 mM PIPES pH 7.4), 0.5% NP40, 12U RNase Inhibitor (Roche) and 100 ng/ml Cycloheximide (Sigma). To separate polysomal RNA from non-polysomal RNA, linear sucrose gradients were built from polysome buffer with 0% to 50% sucrose concentration. Stability of linear gradients was proved with a refractometer (Type MHRB 90; Müller). Cell lysate was cooled on ice and layered onto gradients which were subjected to centrifugation at 37,500 rpm (28,000 × g) in a SW40 rotor (Beckmann Optima™ L Preparative Ultracentrifuge) at 4°C for 120 min. 13 fractions of 1 ml were collected from the top of the gradient. From 600 μl of each fraction RNA was isolated and the ratio of 28S to 18S rRNA was measured to obtain the polysome profile on Agilent 2100 Bioanalyzer [10]. Based on the polysome profile, 200 μl of each selected gradient fraction were used for pooling of samples into polysomal/non-polysomal parts (Additional file 4). An appropriate amount of viral RNA (One Color Spike-In Kit; Agilent Technologies; Cat. No. 5188-5279) for 1 μg of test-RNA was added to allow for the comparison of relative RNA amounts in later analysis. Total RNA was extracted by using the Trizol Reagent (Invitrogen). Subsequently to RNA isolation, a DNase I digest was performed. RNA were checked for quantity, purity and integrity of the 18S and 28S ribosomal bands by capillary electrophoresis using the Agilent 2100 bioanalyzer and the NanoDropND-1000 UV-VIS Spectrophotometer version 3.2.1.

### Microarray analysis

Microarrays were done using the "Low RNA Input linear Amplification Kit Plus, One Color" protocol (Agilent Technologies, Inc. 2007; Cat. N°: 5188-5339) following the manufacturer's standard protocol. Global gene expression analysis was applied in triplicates using mouse expression arrays (Agilent Technologies; Cat. No. G4122F; 41,000 genes represented). Quantity and efficiency of the labeled amplified cRNA were determined using the NanoDrop ND-1000 UV-VIS Spectrophotometer version 3.2.1. The hybridizations were performed for 17 hours at 10 rpm and 65°C in the Hybridization Oven (Agilent). Washing and staining of the arrays were done according to the manufacturer's recommendation. Cy3 intensities were detected by one-color scanning using an Agilent DNA microarray scanner (G2505B) at 5 micron resolution. Scanned image files were visually inspected for artefacts and then analyzed [14].

### Statistics

Total translation activity was calculated comparing mRNA content of free mRNA fractions versus polysomal fractions using one-sided t-test.

For MA analysis, quality control, normalisation and analysis of differentially expressed genes was performed using the software R [54]. The first step of microarray analysis focused on quality control including correlation investigation, hierarchical clustering and principal component analysis. Normalisation was based on the spike-ins, relating expression data to their respective values while preserving the range of data. With regard to differential expression we were interested in significant differences between the time group fold changes of the polysomal (p_6_-p_0_) and non-polysomal (np_6_-np_0 _) fraction, where p_6 _(p_0_) denotes the log2 signal intensity of polysomal RNA at day 0 + 6 h (+0 h), and analogously np marks the non-polysomal fraction. To detect those differences the empirical Bayes statistic of the limma package [55] was applied implying amoderated gene-by-gene t-test followed by p-value adjustment via multiple testing correction according to the Benjamini-Hochberg method [56]. Clustering of the expression profiles as well as sorting into functional and structural groups was done with PantherDB [57]. Information about candidate genes was collected in PubMed searches. Data were submitted to GEO (accession number GSE29744).

### Quantitative Real Time-PCR (q-PCR)

RNA (300 ng/20 μl) was reverse-transcribed using the High Capacity cDNA Reverse Transcription Kit (ABI). 1 μl of the RT reaction mixture was used for quantitative PCR. Primers to amplify *eIF4B, IMPDH2, RPL27a, UBE2k/HIP2, UCP2 *and *β*Actin were purchased from Sigma Aldrich. q-PCRs were performed with 2x Power Sybr Green Mastermix (ABI) and Mx4000™ Multiplex Quantitative PCR System (Stratagene), all samples were assayed in triplicate. Data was analysed using the ΔΔC_T _method with normalizers *HIP2, UCP2 *and *βActin *[58]. The resulting ΔΔC_T _values per replicate are interpretable as the aforementioned fold change of time ratios between fraction groups. One sample t-tests were conducted per gene to test whether the mean of the replicates differs significantly from zero. Above that an analysis of variance was applied to a linear model of the ΔC_T _values to treat influences of time and fraction groups separately [59] (Additional file 6).

### Western Blotting

Total protein from 3T3-L1 cells at time points T0 and T6 was extracted with protein extraction buffer (50 mM NaF, 50 mM Tris HCl, 5 mM NaPPi, 150 mM NaCl, 1% NP-40, 1 mM Na3VO4 and 1 mM EDTA). For each sample, 30 μg protein was denatured in Laemmli Buffer for 5 min at 95°C. Proteins were separated by SDS-PAGE and transferred to PVDF membrane (Millipore) using a tank blot system (Bio-Rad). The membrane was blocked for 1 h in blocking buffer (5% skim milk powder in PBS). After washing three times for 15 min in PBS, the membrane was incubated for 30 min at room temperature with polyclonal antibody against *β*Actin (ab75186; Abcam) in a dilution 1:15,000 in milk (PBS, 2% skim milk powder, 10% fetal bovine serum). The membrane was washed again and incubated with either polyclonal antibodies against eIF4B (ab59300, 1: 2,000), IMPDH2 (ab75790, 1:1,000), UBE2k/HIP2 (ab82950, 1:1,000) or RPL27a (ab74731, 1:1,300) over night at 4°C and afterwards 1 h at room temperature. Goat polyclonal anti-rabbit IgG conjugated with horseradish peroxidase (ab6721, Abcam) was added to the membrane after washing and incubated for 90 min at room temperature. Washed membrane was incubated in Luminata Classico Chemiluminescence Detection Reagent (Millipore) for 2 min and exposed to photo film (ECL, Amersham Biosciences). Changes in protein expression were quantified by densitometry with Image J and normalized with appropriate expression data of *β*Actin. For each of the three biological and two technical replicates per gene the normalized value for time point 6 was related to the one of time point 0. The resulting three ratios per technical replicate were averaged leading to one value per biological replicate. As the Image J measurement itself was additionally repeated threefold, the aforementioned procedure was applied three times and the mean was taken per biological replicate which finally lead to one averaged value per biological replicate. One sample t-tests were conducted per gene to test whether the mean of those 3 values differs significantly from one (Figure 2).

## Competing interests

The authors declare that they have no competing interests.

## Authors' contributions

CF-D performed most of the experimental part including cell culture, RNA isolation, gradients, qPCR, Western blotting and writing of the manuscript. SvdH did statistical analysis under supervision of TB. OL did cell culture and established gradients. GS developed the spike-in strategy used in this project. BB co-developed the strategy. TB participated in the planning of MA, spike-ins, and developed the strategy for the statistical analysis of the MA data. BGB developed the project, supervised the practical part, coordinated the project and wrote the manuscript. All authors read and approved the final manuscript.

## Supplementary Material

Additional file 1 **Microscopical control of adipogenesis**. In comparison to the fibroblastic phenotype of 3T3-L1 preadipocytes (left picture), mature adipocytes´ phenotype (right picture) is round and cells accumulate lipid droplets in the cytoplasm. 3T3-L1 cells two days before hormonal induction (left picture) were stained with *Coomassie blue*. Cells nine days after hormonal induction (right picture) were stained with *Oil Red O *(400× magnification). Click here for file

Additional file 2 **Molecular control of adipogenesis**. Analysis of mRNA steady state levels of *C/EBPβ *(empty boxes) and *PPARγ *(filled boxes) by means of q-PCR in total RNA at time points 0, 0 + 6 h and 9 days. Ct-values were calibrated to day 0, normalized with *β*Actin, (mean of 3 experiments with 3 replicates each, n = 9). *C/EBPβ *was up-regulated 3times at 0 + 6 h and back to base levels at day 9. *PPARγ *was up-regulated at day 9, no significant change of mRNA steady state levels were detected at day 0 + 6 h. Standard deviations are shown by error bars. Click here for file

Additional file 3 **Control of stability of gradients**. Stability of 14 linear gradients was proved with a refractometer. Gradient fractions were collected from top of gradient and percentage of sucrose content was measured. Standard deviations are shown by error bars. Click here for file

Additional file 4 **Sucrose gradient analysis**. Polysomal RNA (28S/18S ratio ~ 2) was separated from non-polysomal RNA (28S/18S ratio ≠2) by sucrose gradient centrifugation. Ratio of 18S and 28S rRNA was measured to obtain the polysome profile on Agilent 2100 Bioanalyzer. Fractions 5 to 7 contain non-polysomal RNA and fractions 9 to 11 polysomal RNA. Click here for file

Additional file 5 **Further information to Table **1. Gene functions, GenBank accession numbers and fold change for mRNAs that are fourfold and greater up- or down-regulated 6 hours after stimulation of adipogenesis. Click here for file

Additional file 6 **Heatmap forhousekeeper-normalized PCR data**. Column 1 to 4 shows housekeeper-normalized values for polysomal (p) and non-polysomal (np) fractions at two time points (0 h and 6 h after hormonal induction). Column 5 shows fraction to time ratio (log; (p_6_-np_6_) - (p_0_-np_0_)). Click here for file

Additional file 7 **Results of Cluster analysis by PANTHER DB**. This Table contains the name of the PANTHER classification category, the genes that map to the respective category, the expected number of genes in the respective category based on the reference genome, plus or minus signs indicating over- or under-representation of the respective category in the experiment and finally the p-values determined by the binomial statistic according to [60]. Click here for file

## References

[B1] Spalding KL Arner E Westermark PO Bernard S Buchholz BA Bergmann O Blomqvist L Hoffstedt J Näslund E Britton T Concha H Hassan M Rydén M Frisén J Arner P Dynamics of fat cell turnover in humans Nature 2008 453 783 787 18454136 10.1038/nature06902

[B2] MacDougald OA Lane MD Transcriptional regulation of gene expression during adipocyte differentiation Annu Rev Biochem 1995 64 345 373 7574486 10.1146/annurev.bi.64.070195.002021

[B3] Mishra A Zhu X Ge K Cheng S-Y Adipogenesis is differentially impaired by thyroid hormone receptor mutant isoforms J Mol Endocrinol 2010 44 247 255 20080985 10.1677/JME-09-0137 PMC3464097

[B4] Poulos SP Dodson MV Hausman GJ Cell line models for differentiation: preadipocytes and adipocytes Exp Biol Med 2010 235 1185 1193 10.1258/ebm.2010.010063 20864461

[B5] Burton GR Guan Y Nagarajan R McGehee RE Jr Microarray analysis of gene expression during early adipocyte differentiation Gene 2002 293 21 31 12137940 10.1016/s0378-1119(02)00726-6

[B6] Rosen ED Hsu C-H Wang X Sakai S Freeman MW Gonzalez FJ Spiegelman BM C/EBPalpha induces adipogenesis through PPARgamma: a unified pathway Genes Dev 2002 16 22 26 11782441 10.1101/gad.948702 PMC155311

[B7] Calkhoven CF Müller C Leutz A Translational control of C/EBPalpha and C/EBPbeta isoform expression Genes Dev 2000 14 1920 1932 10921906 PMC316813

[B8] Kawagishi H Wakoh T Uno H Maruyama M Moriya A Morikawa S Okano H Sherr CJ Takagi M Sugimoto M Hzf regulates adipogenesis through translational control of C/EBP[alpha] EMBO J 2008 27 1481 1490 18418387 10.1038/emboj.2008.76 PMC2396393

[B9] Melamed D Arava Y Genome-wide analysis of mRNA polysomal profiles with spotted DNA microarrays Meth Enzymol 2007 431 177 201 17923236 10.1016/S0076-6879(07)31010-0

[B10] Parent R Beretta L Translational control plays a prominent role in the hepatocytic differentiation of HepaRG liver progenitor cells Genome Biol 2008 9 R19 18221535 10.1186/gb-2008-9-1-r19 PMC2395229

[B11] Melamed D Eliyahu E Arava Y Exploring translation regulation by global analysis of ribosomal association Methods 2009 48 301 305 19426805 10.1016/j.ymeth.2009.04.020

[B12] Carlage T Hincapie M Zang L Lyubarskaya Y Madden H Mhatre R Hancock WS Proteomic profiling of a high-producing Chinese hamster ovary cell culture Anal Chem 2009 81 7357 7362 19663468 10.1021/ac900792z

[B13] Bezy O Vernochet C Gesta S Farmer SR Kahn CR TRB3 blocks adipocyte differentiation through the inhibition of C/EBPbeta transcriptional activity Mol Cell Biol 2007 27 6818 6831 17646392 10.1128/MCB.00375-07 PMC2099230

[B14] Opitz L Salinas-Riester G Grade M Jung K Jo P Emons G Ghadimi BM Beissbarth T Gaedcke J Impact of RNA degradation on gene expression profiling BMC Med Genomics 2010 3 36 20696062 10.1186/1755-8794-3-36 PMC2927474

[B15] Schuirmann D On hypothesis testing to determine if the mean of a normal distribution is contained in a known interval 1981 37 617

[B16] Westlake W Bioequivalence testing - a need to rethink 1981 37 589 594

[B17] Mikulits W Pradet-Balade B Habermann B Beug H Garcia-Sanz JA Müllner EW Isolation of translationally controlled mRNAs by differential screening FASEB J 2000 14 1641 1652 10928999 10.1096/fj.14.11.1641

[B18] Kheterpal I Ku G Coleman L Yu G Ptitsyn AA Floyd ZE Gimble JM Proteome of Human Subcutaneous Adipose Tissue Stromal Vascular Fraction Cells versus Mature Adipocytes Based on DIGE J Proteome Res 2011 10 1519 1527 21261302 10.1021/pr100887r PMC3070065

[B19] Welsh GI Griffiths MR Webster KJ Page MJ Tavaré JM Proteome analysis of adipogenesis Proteomics 2004 4 1042 1051 15048985 10.1002/pmic.200300675

[B20] Lee H-K Lee B-H Park S-A Kim C-W The proteomic analysis of an adipocyte differentiated from human mesenchymal stem cells using two-dimensional gel electrophoresis Proteomics 2006 6 1223 1229 16421933 10.1002/pmic.200500385

[B21] Choi K-L Wang Y Tse CA Lam KSL Cooper GJS Xu A Proteomic analysis of adipocyte differentiation: Evidence that alpha2 macroglobulin is involved in the adipose conversion of 3T3 L1 preadipocytes Proteomics 2004 4 1840 1848 15174150 10.1002/pmic.200300697

[B22] Adachi J Kumar C Zhang Y Mann M In-depth analysis of the adipocyte proteome by mass spectrometry and bioinformatics Mol Cell Proteomics 2007 6 1257 1273 17409382 10.1074/mcp.M600476-MCP200

[B23] Newton BW Cologna SM Moya C Russell DH Russell WK Jayaraman A Proteomic Analysis of 3T3-L1 Adipocyte Mitochondria during Differentiation and Enlargement Journal of Proteome Research 2011 10.1021/pr200491h 21815628

[B24] Molina H Yang Y Ruch T Kim J-W Mortensen P Otto T Nalli A Tang Q-Q Lane MD Chaerkady R Pandey A Temporal profiling of the adipocyte proteome during differentiation using a five-plex SILAC based strategy J Proteome Res 2009 8 48 58 18947249 10.1021/pr800650r PMC2642533

[B25] Qiu Z Wei Y Chen N Jiang M Wu J Liao K DNA synthesis and mitotic clonal expansion is not a required step for 3T3-L1 preadipocyte differentiation into adipocytes J Biol Chem 2001 276 11988 11995 11278974 10.1074/jbc.M011729200

[B26] Lai M-D Xu J Ribosomal Proteins and Colorectal Cancer Curr Genomics 2007 8 43 49 18645623 10.2174/138920207780076938 PMC2474683

[B27] Du J Shi Y Pan Y Jin X Liu C Liu N Han Q Lu Y Qiao T Fan D Regulation of multidrug resistance by ribosomal protein l6 in gastric cancer cells Cancer Biol Ther 2005 4 242 247 15846068 10.4161/cbt.4.2.1477

[B28] Gou Y Shi Y Zhang Y Nie Y Wang J Song J Jin H He L Gao L Qiao L Wu K Fan D Ribosomal protein L6 promotes growth and cell cycle progression through upregulating cyclin E in gastric cancer cells Biochem Biophys Res Commun 2010 393 788 793 20171175 10.1016/j.bbrc.2010.02.083

[B29] Obregon M-J Thyroid hormone and adipocyte differentiation Thyroid 2008 18 185 195 18279019 10.1089/thy.2007.0254

[B30] Lu C Cheng S-Y Thyroid hormone receptors regulate adipogenesis and carcinogenesis via crosstalk signaling with peroxisome proliferator-activated receptors J Mol Endocrinol 2010 44 143 154 19741045 10.1677/JME-09-0107 PMC3464095

[B31] Kumar KU Srivastava SP Kaufman RJ Double-Stranded RNA-Activated Protein Kinase (PKR) Is Negatively Regulated by 60S Ribosomal Subunit Protein L18 Mol Cell Biol 1999 19 1116 1125 9891046 10.1128/mcb.19.2.1116 PMC116041

[B32] Yamada T Sasaki S Sukegawa S Miyake T Fujita T Kose H Morita M Takahagi Y Murakami H Morimatsu F Sasaki Y Association of a single nucleotide polymorphism in ribosomal protein L27a gene with marbling in Japanese Black beef cattle Anim Sci J 2009 80 631 635 20163651 10.1111/j.1740-0929.2009.00688.x

[B33] Caldarola S De Stefano MC Amaldi F Loreni F Synthesis and function of ribosomal proteins - fading models and new perspectives FEBS Journal 2009 276 3199 3210 19438715 10.1111/j.1742-4658.2009.07036.x

[B34] Van den Broeke A Van Poucke M Marcos-Carcavilla A Hugot K Hayes H Bertaud M Van Zeveren A Peelman L Characterization of the ovine ribosomal protein SA gene and its pseudogenes BMC Genomics 2010 11 179 20233419 10.1186/1471-2164-11-179 PMC2850357

[B35] Ku H-C Chang H-H Liu H-C Hsiao C-H Lee M-J Hu Y-J Hung P-F Liu C-W Kao Y-H Green tea (-)-epigallocatechin gallate inhibits insulin stimulation of 3T3-L1 preadipocyte mitogenesis via the 67-kDa laminin receptor pathway Am J Physiol, Cell Physiol 2009 297 121 132 10.1152/ajpcell.00272.2008 19176763

[B36] Kao Y-H Chang H-H Lee M-J Chen C-L Tea, obesity, and diabetes Mol Nutr Food Res 2006 50 188 210 16416476 10.1002/mnfr.200500109

[B37] Ruggero D Sonenberg N The Akt of translational control Oncogene 2005 24 7426 7434 16288289 10.1038/sj.onc.1209098

[B38] Rui L A link between protein translation and body weight J Clin Invest 2007 117 310 313 17273554 10.1172/JCI31289 PMC1783829

[B39] Topisirovic I Sonenberg N Translational control by the eukaryotic ribosome Cell 2011 145 333 334 21529706 10.1016/j.cell.2011.04.006

[B40] Wang X Proud CG The mTOR pathway in the control of protein synthesis Physiology (Bethesda) 2006 21 362 369 16990457 10.1152/physiol.00024.2006

[B41] Hernández G Vazquez-Pianzola P Functional diversity of the eukaryotic translation initiation factors belonging to eIF4 families Mechanisms of Development 2005 122 865 876 15922571 10.1016/j.mod.2005.04.002

[B42] Clemens MJ Bushell M Jeffrey IW Pain VM Morley SJ Translation initiation factor modifications and the regulation of protein synthesis in apoptotic cells Cell Death Differ 2000 7 603 615 10889505 10.1038/sj.cdd.4400695

[B43] Shahbazian D Parsyan A Petroulakis E Topisirovic I Martineau Y Gibbs BF Svitkin Y Sonenberg N Control of Cell Survival and Proliferation by Mammalian Eukaryotic Initiation Factor 4B Mol Cell Biol 2010 30 1478 1485 20086100 10.1128/MCB.01218-09 PMC2832492

[B44] Zhang HH Huang J Düvel K Boback B Wu S Squillace RM Wu C-L Manning BD Insulin Stimulates Adipogenesis through the Akt-TSC2-mTORC1 Pathway PLoS ONE 2009 4 e6189 19593385 10.1371/journal.pone.0006189 PMC2703782

[B45] Grandori C Cowley SM James LP Eisenman RN The Myc/Max/Mad network and the transcriptional control of cell behavior Annu Rev Cell Dev Biol 2000 16 653 699 11031250 10.1146/annurev.cellbio.16.1.653

[B46] Freytag SO Geddes TJ Reciprocal regulation of adipogenesis by Myc and C/EBP alpha Science 1992 256 379 382 1566086 10.1126/science.256.5055.379

[B47] Heath VJ Gillespie DA Crouch DH Inhibition of the terminal stages of adipocyte differentiation by cMyc Exp Cell Res 2000 254 91 98 10623469 10.1006/excr.1999.4736

[B48] Kim S-J Lee K-H Lee Y-S Mun E-G Kwon D-Y Cha Y-S Transcriptome analysis and promoter sequence studies on early adipogenesis in 3T3-L1 cells Nutr Res Pract 2007 1 19 28 20535381 10.4162/nrp.2007.1.1.19 PMC2882572

[B49] Dai M-S Lu H Crosstalk between c-Myc and ribosome in ribosomal biogenesis and cancer J Cell Biochem 2008 105 670 677 18773413 10.1002/jcb.21895 PMC2569974

[B50] Fensterl V Sen GC The ISG56/IFIT1 gene family J Interferon Cytokine Res 2011 31 71 78 20950130 10.1089/jir.2010.0101 PMC3021354

[B51] Xu G Li Y An W Zhang W Ghrelin and cell differentiation Acta Biochim Biophys Sin (Shanghai) 2008 40 841 847 18850048

[B52] Liu J Lin H Cheng P Hu X Lu H Effects of ghrelin on the proliferation and differentiation of 3T3-L1 preadipocytes J Huazhong Univ Sci Technol Med Sci 2009 29 227 230 19399410 10.1007/s11596-009-0218-x

[B53] Zhang W Zhao L Lin TR Chai B Fan Y Gantz I Mulholland MW Inhibition of adipogenesis by ghrelin Mol Biol Cell 2004 15 2484 2491 15034137 10.1091/mbc.E03-09-0657 PMC404039

[B54] Team RDC R: A Language and Environment for Statistical Computing 1 ISBN 3- 900051-07-0

[B55] Smyth GK Linear Models and Empirical Bayes Methods for Assessing Differential Expression in Microarray Experiments Statistical Applications in Genetics and Molecular Biology 2004 3 1 25 10.2202/1544-6115.1027 16646809

[B56] Benjamini Y Hochberg Y Controlling the False Discovery Rate: A Practical and: Powerful Approach to Multiple Testing Journal of the Royal Statistical Society Series B (Methodological) 1995 57 289 300

[B57] Thomas PD Campbell MJ Kejariwal A Mi H Karlak B Daverman R Diemer K Muruganujan A Narechania A PANTHER: A Library of Protein Families and Subfamilies Indexed by Function Genome Research 2003 13 2129 2141 12952881 10.1101/gr.772403 PMC403709

[B58] Schmittgen TD Livak KJ Analyzing real-time PCR data by the comparative CT method Nat Protocols 2008 3 1101 1108 10.1038/nprot.2008.73 18546601

[B59] Chambers J Freeny A Heiberger R Analysis of variance; designed experiments Statistical Models in S 1992 Pacific Grove, California: Wadsworth & Brooks/Cole

[B60] Cho RJ Campbell MJ Transcription, genomes, function Trends Genet 2000 16 409 415 10973070 10.1016/s0168-9525(00)02065-5

[B61] Zheng S Yao Y Dong Y Lin F Zhao H Shen Z Sun Y Tang L Down-regulation of ribosomal protein L7A in human osteosarcoma J Cancer Res Clin Oncol 2009 135 1025 1031 19125294 10.1007/s00432-008-0538-4 PMC12160200

