# Supplementary figures and images for: Novel polysome messages and changes in translational activity appear after induction of adipogenesis in 3T3-L1 cells
Source: BMC Mol Biol. 2012 Mar 21;13:9. doi: 10.1186/1471-2199-13-9 (PMC3347988; doi:10.1186/1471-2199-13-9)

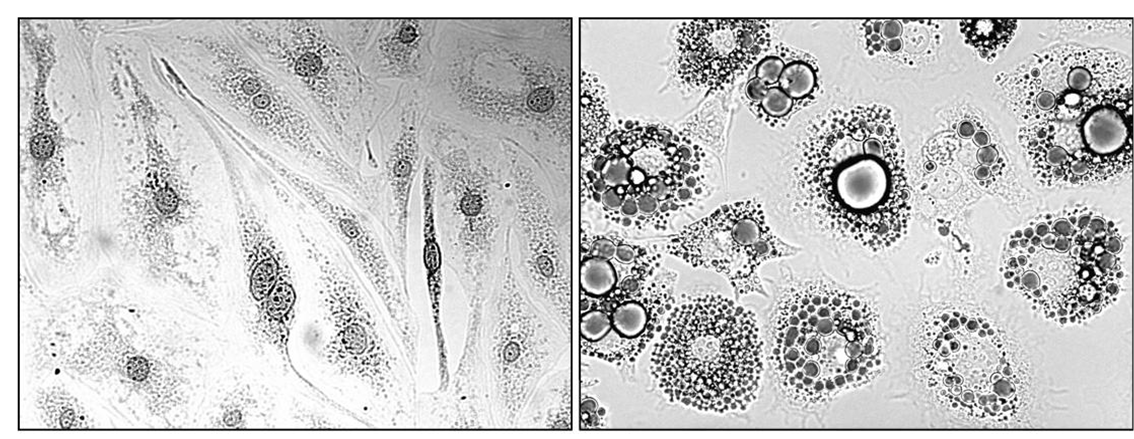

Supplement: Additional file 1 — Microscopical control of adipogenesis. In comparison to the fibroblastic phenotype of 3T3-L1 preadipocytes (left picture), mature adipocytes´ phenotype (right picture) is round and cells accumulate lipid droplets in the cytoplasm. 3T3-L1 cells two days before hormonal induction (left picture) were stained with Coomassie blue. Cells nine days after hormonal induction (right picture) were stained with Oil Red O (400× magnification). [file 1471-2199-13-9-S1.TIFF]

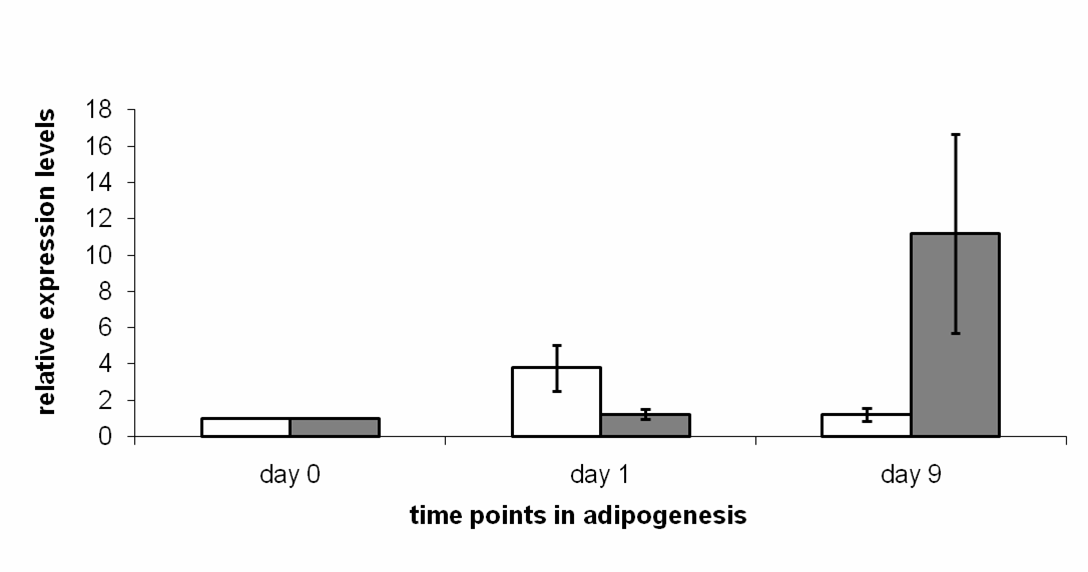

Supplement: Additional file 2 — Molecular control of adipogenesis. Analysis of mRNA steady state levels of C/EBPβ (empty boxes) and PPARγ (filled boxes) by means of q-PCR in total RNA at time points 0, 0 + 6 h and 9 days. Ct-values were calibrated to day 0, normalized with βActin, (mean of 3 experiments with 3 replicates each, n = 9). C/EBPβ was up-regulated 3times at 0 + 6 h and back to base levels at day 9. PPARγ was up-regulated at day 9, no significant change of mRNA steady state levels were detected at day 0 + 6 h. Standard deviations are shown by error bars. [file 1471-2199-13-9-S2.TIFF]

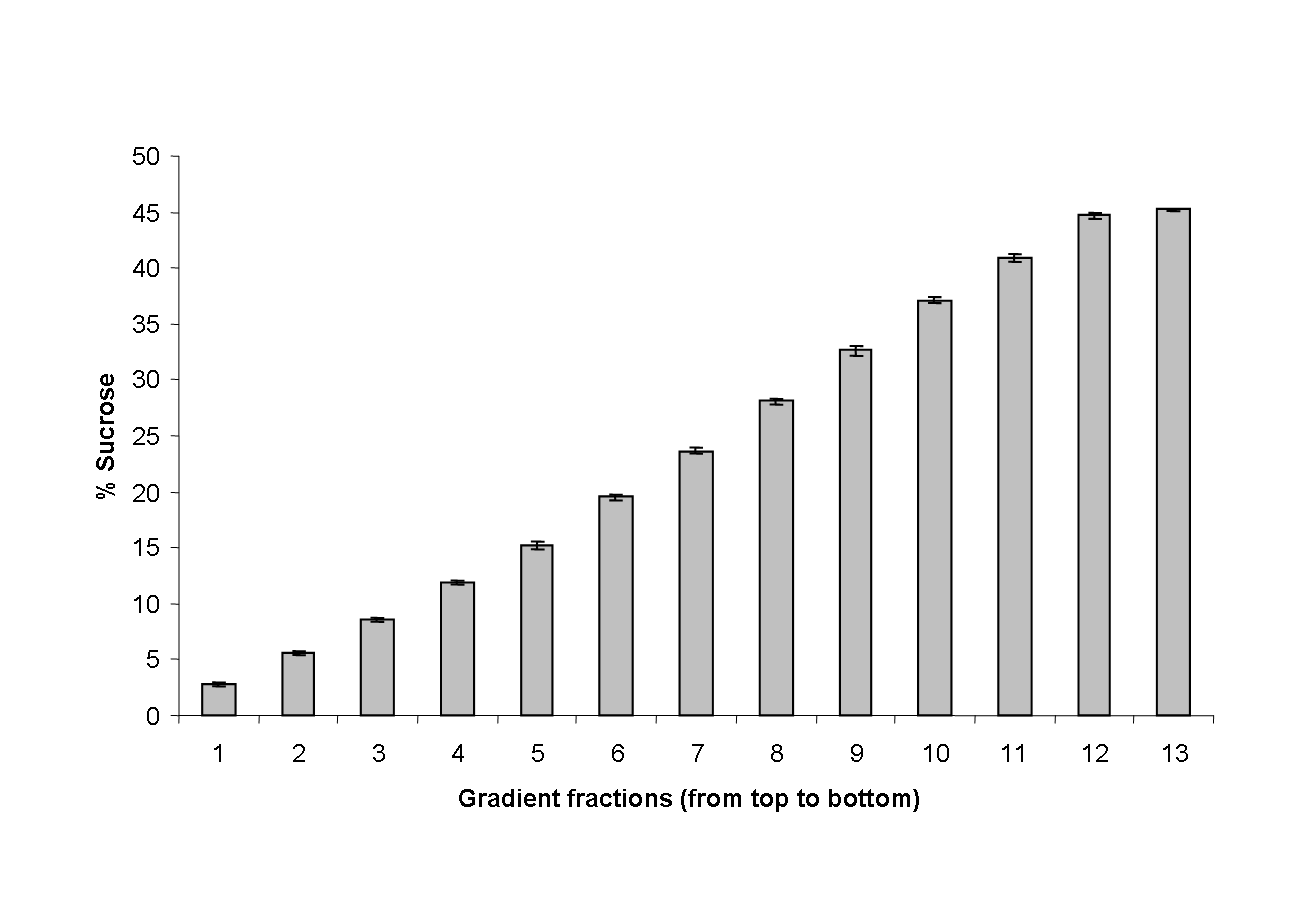

Supplement: Additional file 3 — Control of stability of gradients. Stability of 14 linear gradients was proved with a refractometer. Gradient fractions were collected from top of gradient and percentage of sucrose content was measured. Standard deviations are shown by error bars. [file 1471-2199-13-9-S3.TIFF]

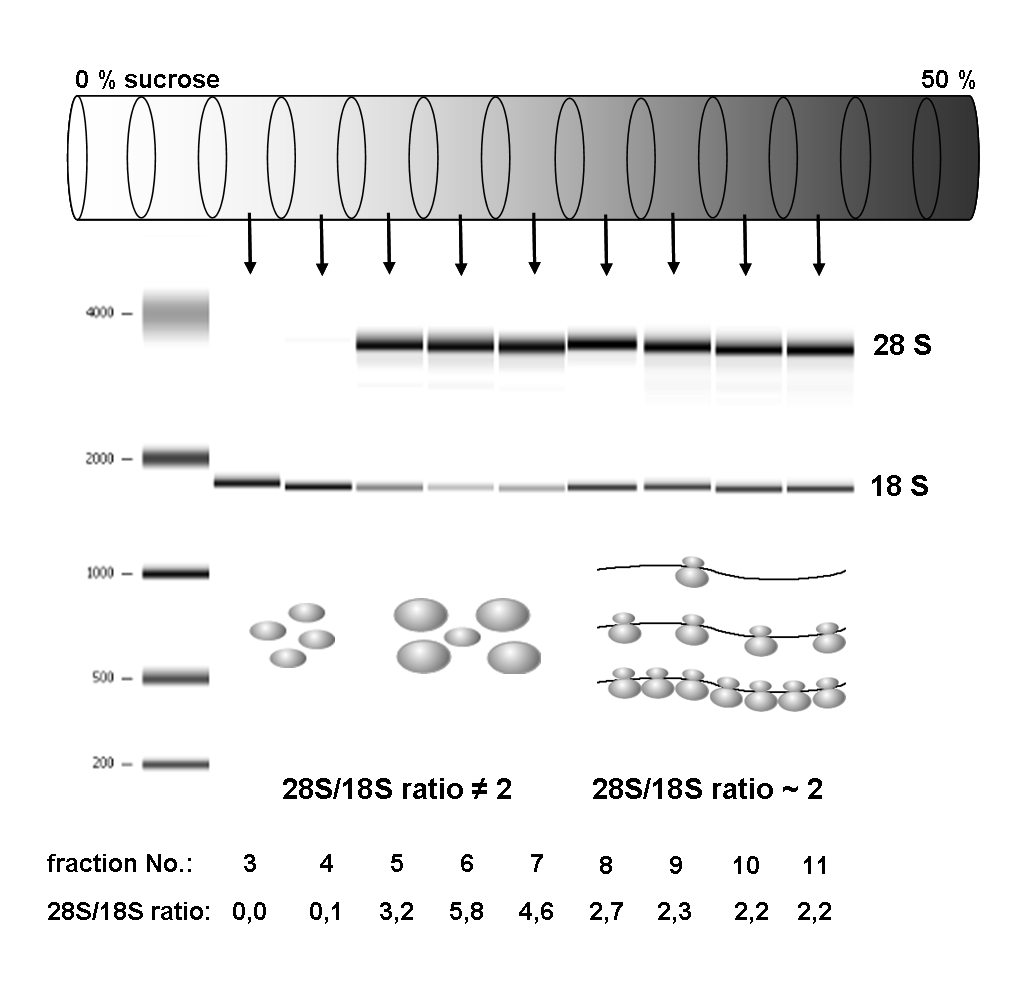

Supplement: Additional file 4 — Sucrose gradient analysis. Polysomal RNA (28S/18S ratio ~ 2) was separated from non-polysomal RNA (28S/18S ratio ≠2) by sucrose gradient centrifugation. Ratio of 18S and 28S rRNA was measured to obtain the polysome profile on Agilent 2100 Bioanalyzer. Fractions 5 to 7 contain non-polysomal RNA and fractions 9 to 11 polysomal RNA. [file 1471-2199-13-9-S4.TIFF]

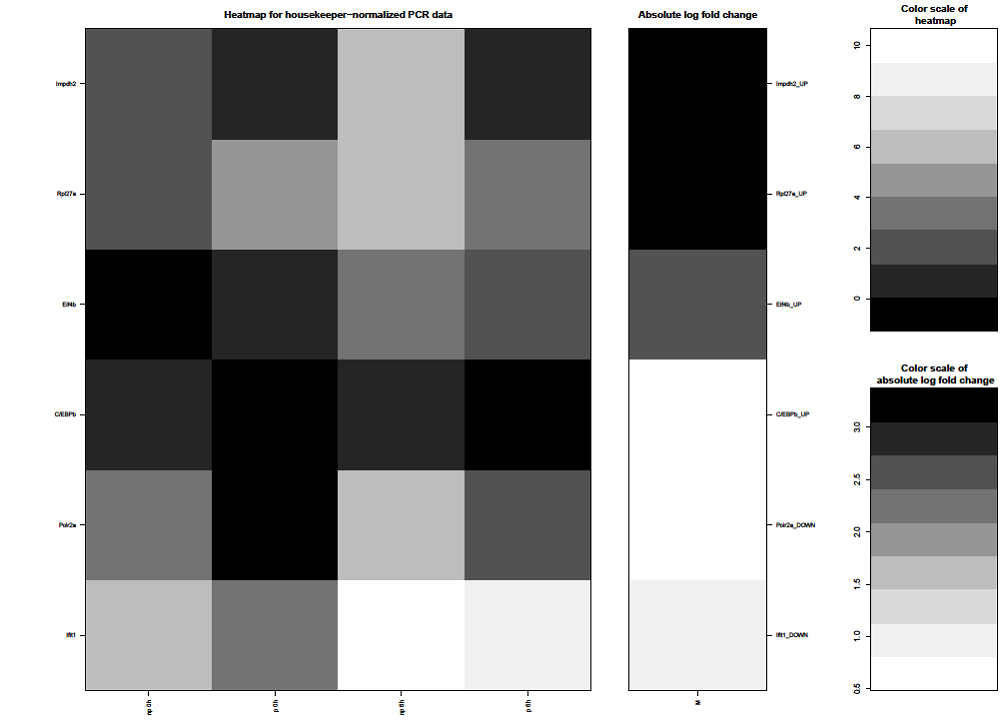

Supplement: Additional file 6 — Heatmap forhousekeeper-normalized PCR data. Column 1 to 4 shows housekeeper-normalized values for polysomal (p) and non-polysomal (np) fractions at two time points (0 h and 6 h after hormonal induction). Column 5 shows fraction to time ratio (log; (p6-np6) - (p0-np0)). [file 1471-2199-13-9-S6.TIFF]
