# Supplementary material for: Novel polysome messages and changes in translational activity appear after induction of adipogenesis in 3T3-L1 cells
Source: BMC Mol Biol. 2012 Mar 21;13:9. doi: 10.1186/1471-2199-13-9 (PMC3347988; doi:10.1186/1471-2199-13-9)
Supplement: Additional file 5 — Further information to Table 1. Gene functions, GenBank accession numbers and fold change for mRNAs that are fourfold and greater up- or down-regulated 6 hours after stimulation of adipogenesis. [file 1471-2199-13-9-S5.PDF]

Additional file 5 - Further information to Table 1
Gene functions, GenBank accession numbers and fold change for mRNAs that are fourfold and greater up- or down-regulated 6 hours after stimulation of adipogenesis.

Description	Symbol	Gene functions	GenBank	fold change	fdr
Genes up-regulated 6h after hormonal induction					
interferon regulatory factor 6	*IRF6*	nucleobase, nucleoside, nucleotide and nucleic, acid metabolic process, immune response, response to interferon-gamma	AK087631	14.22	1.78E-03
polymerase (RNA) II (DNA directed) polypeptide A	*POLR2a*	nucleobase, nucleoside, nucleotide and nucleic acid metabolic process	NM_009089	13.23	1.07E-03
pleckstrin homology domain containing, family N member 1	*PLEKHN1*	cell communication, signal transduction, cellular defense response	NM_001008233	7.80	1.89E-03
proprotein convertase subtilisin/kexin type 1 inhibitor	*PCSK1n*	unclassified	NM_013892	7.56	9.28E-04
ribosomal protein L27a	*RPL27a*	protein metabolic process	NM_011975	7.42	3.83E-04
eukaryotic translation initiation factor 4B	*eIF4B*	protein metabolic process	NM_145625	7.15	3.83E-04
inosine 5'-phosphate dehydrogenase 2	*IMPDH2*	nucleobase, nucleoside, nucleotide and nucleic acid metabolic process	NM_011830	5.97	3.83E-04
lactoperoxidase	*LPO*	oxygen and reactive oxygen species metabolic process, immune response	NM_080420	5.56	9.70E-04
Nicotinamide nucleotide adenylyltransferase 2	*NMNAT2*	nucleobase, nucleoside, nucleotide and nucleic acid metabolic process	NM_175460	5.45	3.83E-04
ribosomal protein S18	*RPS18*	protein metabolic process	NM_011296	5.29	3.83E-04
heat shock protein 8	*HSPA8*	protein metabolic process, response to stress	NM_031165	5.20	3.83E-04
sema domain, immunoglobulin domain (Ig), short basic domain, secreted, (semaphorin) 3G	*SEMA3g*	signal transduction, developmental process, cell surface receptor linked signal transduction, cell adhesion	AK129018	5.19	3.83E-04
family with sequence	*FAM131a*	unclassified	NM_133778	4.97	3.83E-

similarity 131, member A					04
solute carrier family 25, member 30	*SLC25a30*	phosphate, lipid, nucleobase, nucleoside, nucleotide and nucleic acid metabolic process; phosphate, lipid, cation, ion, nucleobase, nucleoside, nucleotide and nucleic acid transport	NM_026232	4.88	3.83E-04
RIKEN cDNA 4930558C23 gene	*4930558C23RIK*	not described	AK006075	4.88	1.22E-03
small inducible cytokine subfamily E, member 1	*SCYE1*	protein metabolic process	NM_007926	4.81	3.83E-04
S-adenosylhomocysteine hydrolase	*AHCY*	nucleobase, nucleoside, nucleotide and nucleic acid metabolic process	L32836	4.80	3.83E-04
vasohibin 1	*VASH1*	unclassified	NM_177354	4.78	3.83E-04
heat shock protein 90 alpha (cytosolic), class B member 1	*HSP90ab1*	protein metabolic process, response to stress	NM_008302	4.75	3.83E-04
ADP-ribosylation factor-like 4D	*ARL4d*	not described	NM_031160	4.69	8.90E-04
myelocytomatosis oncogene	*MYC*	cellular process, induction of apoptosis, cell cycle	NM_010849	4.66	3.95E-04
TSC22 domain family, member 3	*TSC22d3*	nucleobase, nucleoside, nucleotide and nucleic acid metabolic process	AF024519	4.60	4.35E-04
ribosomal protein L7A	*RPL7a*	protein metabolic process; nucleobase, nucleoside, nucleotide and nucleic acid metabolic process	NM_013721	4.54	3.83E-04
solute carrier family 25, member 30	*SLC25a30*	phosphate, lipid, nucleobase, nucleoside, nucleotide and nucleic acid metabolic process; phosphate, lipid, cation, ion, nucleobase, nucleoside, nucleotide and nucleic acid transport	AK159732	4.53	4.35E-04
no description	no symbol	not described	XR_002409	4.48	3.83E-04
solute carrier family 25 (mitochondrial carrier,	*SLC25a5*	phosphate, lipid, nucleobase, nucleoside,	NM_007451	4.45	3.83E-04

adenine nucleotide translocator), member 5		nucleotide and nucleic acid metabolic process; phosphate, lipid, cation, ion, nucleobase, nucleoside, nucleotide and nucleic acid transport			
TBC1 domain family, member 22a	*TBC1d22a*	cellular component organization, exocytosis, cellular component morphogenesis, intracellular protein transport	NM_145476	4.33	3.83E-04
TSC22 domain family, member 3	*TSC22d3*	nucleobase, nucleoside, nucleotide and nucleic acid metabolic process	NM_001077364	4.32	3.83E-04
procollagen-lysine, 2-oxoglutarate 5-dioxygenase 3	*PLOD3*	protein metabolic process, cell adhesion	NM_011962	4.31	4.48E-04
BCL2-associated athanogene 3	*BAG3*	protein metabolic process, signal transduction, negative regulation of apoptosis	AJ250687	4.29	4.95E-04
cyclin-dependent kinase inhibitor 1C (P57)	*CDKN1c*	cellular process cell cycle	NM_009876	4.26	7.35E-04
small proline-rich protein 2A	*SPRR2a*	unclassified	NM_011468	4.23	3.83E-04
heat shock factor 1	*HSF1*	nucleobase, nucleoside, nucleotide and nucleic acid metabolic process; response to stress, immune system process, response to stimulus	Z49206	4.23	6.93E-04
no description	no symbol	not described	NM_001013830	4.23	3.83E-04
ribosomal protein S15a pseudogene	*GM13253*	not described	XM_973351	4.22	3.83E-04
ribosomal protein L18	*RPL18*	protein metabolic process	NM_009077	4.22	3.83E-04
predicted gene, 675507	*675507*	not described	XR_005114	4.10	3.83E-04
ribosomal protein SA	*RPSa*	protein metabolic process, nucleobase, nucleoside, nucleotide and nucleic acid metabolic process	NM_011029	4.09	4.31E-04
dapper homolog 1, antagonist of beta-	*DACT1*	unclassified	NM_021532	4.08	1.19E-03

catenin (xenopus)					
sorting nexin 17	*SNX17*	vesicle-mediated transport, endocytosis, intracellular protein transport	NM_153680	4.03	2.40E-02
dehydrodolichyl diphosphate synthase	*DHDDS*	protein metabolic process	NM_026144	4.01	4.09E-04
ribosomal protein L6	*RPL6*	protein metabolic process	NM_011290	4.00	4.09E-04
ethanolaminephosphotransferase 1	*EPT1*	not described	NM_027652	4.00	4.08E-04
Genes down-regulated 6h after hormonal induction					
interferon-induced protein with tetratricopeptide repeats 1	*IFIT1*	response to interferon gamma immune response, response to stimulus	NM_008331	0.25	4.56E-04
ghrelin	*GHRL*	cell surface receptor linked signal transduction, cell communication	NM_021488	0.16	1.61E-03
