# Supplementary material for: Novel polysome messages and changes in translational activity appear after induction of adipogenesis in 3T3-L1 cells
Source: BMC Mol Biol. 2012 Mar 21;13:9. doi: 10.1186/1471-2199-13-9 (PMC3347988; doi:10.1186/1471-2199-13-9)
Supplement: Additional file 7 — Results of Cluster analysis by PANTHER DB. This Table contains the name of the PANTHER classification category, the genes that map to the respective category, the expected number of genes in the respective category based on the reference genome, plus or minus signs indicating over- or under-representation of the respective category in the experiment and finally the p-values determined by the binomial statistic according to [60]. [file 1471-2199-13-9-S7.PDF]

Additional file 7 - Results of Cluster analysis by PANTHER DB.

This Table contains the name of the PANTHER classification category, the genes that map to the respective category, the expected number of genes in the respective category based on the reference genome, plus or minus signs indicating over- or under-representation of the respective category in the experiment and finally the p-values determined by the binomial statistic according to [61].

PANTHER classification	*genes*	expected	over/under	P-value
Molecular Function of up-regulated genes 6h after hormonal induction				
nucleic acid binding	*eIF4B, HSF1, IRF6, MYC, POLR2a, RPL18, RPL27a, RPL6, RPL7a, RPS18, RPSa, TSC22d3*	6.03	+	1.13E-02
structural constituent of ribosome	*RPL18, RPL27a, RPL6, RPL7a, RPS18, RPSa*	0.68	+	5.57E-05
translation initiation factor activity	*eIF4B*	0.14	+	1.29E-01
transcription regulator activity	*HSF1, IRF6, MYC, TSC22d3*	2.86	+	3.20E-01
nucleotidyltransferase activity	*NMNAT2, POLR2a*	0.27	+	2.99E-02
amino acid transmembrane transporter activity	*SLC25a5, SLC25a30*	0.14	+	8.92E-03
kinase inhibitor activity	*CDKN1c*	0.17	+	1.53E-01
aminoacyl-tRNA ligase activity	*SCYE1*	0.05	+	4.70E-02
oxidoreductase activity	*IMPDH2, LPO, PLOD3*	1.13	+	1.03E-01
Protein Class of up-regulated genes 6h after hormonal induction				
ribosomal protein	*RPL18, RPL27a, RPL6, RPL7a, RPS18, RPSa*	0.67	+	4.78E-05
RNA binding protein	*eIF4B, RPL18, RPL27a, RPL6, RPL7a, RPS18, RPSa*	1.50	+	6.18E-04
chaperone	*BAG3, HSPA8, HSP90ab1*	0.18	+	7.27E-04
nucleotidyltransferase	*NMNAT2, POLR2a*	0.13	+	7.38E-03

amino acid transporter	*SLC25a5, SLC25a30*	0.14	+	8.92E-03
kinase inhibitor	*CDKN1c*	0.02	+	2.12E-02
aminoacyl-tRNA synthetase	*SCYE1*	0.03	+	2.64E-02
peroxidase	*LPO*	0.04	+	3.80E-02
oxidoreductase	*IMPDH2, LPO, PLOD3*	0.91	+	6.26E-02
translation factor	*eIF4B*	0.10	+	9.55E-02
acyltransferase	*DHDDS*	0.11	+	1.05E-01
oxygenase activity	*PLOD3*	0.12	+	1.12E-01
hydrolase	*AHCY, TBC1d22a*	3.34	-	3.39E-01
basic helix-loop-helix transcription factor	*MYC*	0.16	+	1.45E-01
dehydrogenase	*IMPDH2*	0.35	+	2.98E-01
transcription factor	*HSF1, IRF6, MYC, TSC22d3*	2.82	+	3.10E-01
structural protein	*SPRR2a*	0.40	+	3.29E-01
signaling molecule	*PLEKHN1, SEMA3g*	1.40	+	4.11E-01
Protein Class of down-regulated gene 6h after hormonal induction				
peptide hormone	*GHRL*	0.01	+	1.32E-02
